# Why the Spectral Radius? An intuition-building introduction to the basic reproduction number

**DOI:** 10.1007/s11538-022-01057-9

**Published:** 2022-08-05

**Authors:** Andrew F. Brouwer

**Affiliations:** Department of Epidemiology, University of Michigan, 1415 Washington Heights, Ann Arbor, MI 48109 USA

**Keywords:** Basic reproduction number, infectious disease model, mathematical epidemiology, next generation matrix, spectral radius

## Abstract

The basic reproduction number ${\mathscr {R}}_0$ is a fundamental concept in mathematical epidemiology and infectious disease modeling. Loosely speaking, it describes the number of people that an infectious person is expected to infect. The basic reproduction number has profound implications for epidemic trajectories and disease control strategies. It is well known that the basic reproduction number can be calculated as the spectral radius of the next generation matrix, but *why* this is the case may not be intuitively obvious. Here, we walk through how the discrete, next generation process connects to the ordinary differential equation disease system of interest, linearized at the disease-free equilibrium. Then, we use linear algebra to develop a geometric explanation of why the spectral radius of the next generation matrix is an epidemic threshold. Finally, we work through a series of examples that help to build familiarity with the kinds of patterns that arise in parameter combinations produced by the next generation method. This article is intended to help new infectious disease modelers develop intuition for the form and interpretation of the basic reproduction number in their disease systems of interest.

## Introduction

The basic reproduction number for epidemic models, denoted ${\mathscr {R}}_0$, is a fundamental concept of mathematical epidemiology. Accordingly, those learning infectious disease modeling need to develop both a technical and intuitive understanding of ${\mathscr {R}}_0$ and be able to both calculate and interpret it for their models. Many students come to infectious disease modeling with varying degrees of epidemiological and mathematical training. There exist many excellent introductions and overviews of ${\mathscr {R}}_0$ (e.g., Diekmann et al. 1990; Dietz 1993; Van Den Driessche and Watmough 2002; Heesterbeek 2002; Roberts 2007; van den Driessche and Watmough 2008; van den Driessche 2017), and this paper does not introduce new mathematical results. Instead, it is intended as an introduction to help new mathematical modelers in epidemiology connect the dots between the two disciplines.

The reader is assumed to have at least an introductory knowledge of differential equations and linear algebra. While a knowledge of linear algebra is not, strictly speaking, necessary to understand, develop, or simulate an epidemic model, it is both convenient for many analyses and necessary for developing a deeper understanding of the modeled disease systems. For students starting to explore infectious disease modeling, it is important to recognize that knowing how to invert and multiply matrices as one might in a basic linear algebra class does not necessarily translate into an understanding of what the quantities mean, and that having a heuristic understanding of the epidemiological concepts is not always enough to be confident in the mathematical techniques. In this paper, we break down the basic reproduction number and develop an understanding of it using basic linear algebra concepts. Then, we examine a series of simple models to understand why common patterns arise in the parameter combinations representing ${\mathscr {R}}_0$ and how to interpret them. Note that this paper focuses on ordinary differential equation (ODE) compartmental infectious disease models; other types of models, e.g., stochastic, partial differential equation, etc., require different, though often related, approaches to calculating and interpreting the basic reproduction number (Allen and Lahodny 2012; Allen and van den Driessche 2013; Magal et al. 2019; Barril et al. 2021).

## The Definition and Importance of the Basic Reproduction Number

The basic reproduction number ${\mathscr {R}}_0$ is defined as the average number of secondary cases arising from a typical primary case in an entirely susceptible population over their infectious lifetime (Diekmann and Heesterbeek 2000; Anderson and May 1992). There are three key pieces to this definition. First, we are considering a single infectious person in an otherwise susceptible population. That means that ${\mathscr {R}}_0$ is only defined at the start of an outbreak, though it impacts the entire epidemic trajectory. Second, we are interested in how many new cases a single case will generate, on average. Any specific person will be responsible for infecting more or fewer new people, but we want to know whether we expect the disease to infect more than 1 person (and thus grow the size of the outbreak) or fewer than 1 (and shrink the size of the outbreak) on average. This threshold value of 1 is important because it represents exact replacement—if each person infected only one person, the outbreak would neither grow nor shrink. The third key piece of the definition is the emphasis on the infectious lifetime of the primary case. A highly infectious disease could have a small ${\mathscr {R}}_0$ if people recover quickly; similarly, a disease that is not very infectious could have a high ${\mathscr {R}}_0$ if people can become chronic shedders. Another intuitive way to think about the ${\mathscr {R}}_0$ is as the product of the “DOTS,” that is, duration, opportunity, transmission probability, and susceptibility (Kucharski 2020). The values of $R_0$ vary greatly by disease, ranging from close to 1 for seasonal influenza to 5–7 for smallpox and polio to 12–18 for measles and pertussis, but are also dependent on some attributes of the population (Fine 1993).

Mathematical modeling is often used to estimate the basic reproductive number for a disease system; in fact, the basic reproduction number is widely considered the most useful contribution of mathematics to epidemiology. Epidemic models are a class of models that represent disease processes—chiefly transmission and recovery, but possibly others—in mathematical form. There are many resources available for those interested in infectious disease modeling more broadly (e.g., Diekmann and Heesterbeek 2000; Anderson and May 1992; Hethcote 2000; Brauer et al. 2008; Vynnycky and White 2010; Keeling and Rohani 2011; Brouwer et al. 2022).

The most basic epidemic model is the ODE, compartmental SIR model (Kermack and McKendrick 1927), which capture the processes of transmission and recovery. The SIR model tracks the numbers of people in a constant population (*N*) who are susceptible (*S*) to disease, who are infectious (*I*), and who have recovered (*R*) over time *t*. This model has two parameters, the transmission rate $\beta$, and the recovery rate $\gamma$. The classic equations are

1 $$\begin{aligned} \begin{aligned} \frac{dS}{dt}&= -\beta (S/N)I,\\ \frac{dI}{dt}&= \beta (S/N)I -\gamma I,\\ \frac{dR}{dt}&= \gamma I. \end{aligned} \end{aligned}$$

Transmission is the more complex of the two processes, as it requires individuals to come together and interact. The transmission rate can be thought of as a contact rate times a per-contact probability of transmitting the infection. But, not all of an infectious individual’s contacts will be susceptible (they could be infectious or recovered instead), so we also account for the time-varying proportion of contacts that are susceptible, *S*/*N*, in the rate. Recovery is a more straightforward process and is modeled as a linear rate $\gamma$. (Note that Eq. (1) is the frequency-dependent form of the SIR equations, so named because the dynamics are independent of the population size *N*; the density-dependent form of the equations, in which the transmission term is given by $\beta ' SI$, is also commonly used and is equivalent for an unchanging population size when $\beta ' = \beta /N$).

In the SIR model, the basic reproduction number is given by the ratio of the transmission rate to the recovery rate $\beta /\gamma$. Why is this the case? At the beginning of the outbreak, $I\approx 1$ and $S\approx N$. Consider the initial infectious person. They will infect $\beta S/N\approx \beta$ people per day on average. How many days are the individual infectious, on average? The formulation of the SIR model as a system of ODEs requires an assumption that the rates are exponential, which implies that the length of the infectious period is exponentially distributed (Greenhalgh and Rozins 2021) (though there are ways to achieve more realistic distributions of the infectious period, e.g., through distributed delays (Lloyd 2001; Krylova and Earn 2013; Hurtado and Kirosingh 2019; Greenhalgh and Rozins 2021)). For an exponentially distributed infectious period, the average duration is one over the rate of leaving the infectious compartment, $1/\gamma$. If an infectious person is infecting $\beta$ new people per day over $1/\gamma$ days, then we expect them to infect $\beta /\gamma$ people over their infectious lifetime. In terms of the “DOTS,” $\beta$ represents the product of opportunity, transmission probability, and susceptibility, while $1/\gamma$ represents duration.

While it was relatively straightforward to build an intuitive argument for this value of ${\mathscr {R}}_0$ for the SIR model, more complicated systems may not have such an immediate way to understand ${\mathscr {R}}_0$. This is particularly true for systems with multiple infection pathways (such as when there is an environmental compartment (Li et al. 2009; Tien and Earn 2010)), with multiple infectious species (such as in the case of vectorborne diseases (Nishiura, et al. 2006)), or with multiple sites of interest on a single person (as may be the case for the human papillomavirus (Brouwer et al. 2015)). In the next section, we will discuss the next generation matrix approach to formally calculating the basic reproduction from mathematical models.

In epidemic models, the basic reproduction number acts as a threshold value that controls the local stability of the disease-free equilibrium: if ${\mathscr {R}}_0<1$, the disease will die off quickly, while if ${\mathscr {R}}_0>1$, the disease will become epidemic. (For those who have studied dynamical systems, this means that ${\mathscr {R}}_0$ is a bifurcation parameter). Although ${\mathscr {R}}_0$ is only defined in the context of a nascent outbreak, its value influences the entire trajectory, including the cumulative incidence of an outbreak, i.e., the fraction of the population that is ever infected in the outbreak (Ma and Earn 2006; Miller 2012). If ${\mathscr {R}}_0$ is small (but still larger than 1), outbreaks are slower, flatter, and longer, infecting fewer people overall; if ${\mathscr {R}}_0$ is large, on the other hand, outbreaks are more explosive, peaking higher and faster, infecting more people overall, and then burning out (Anderson and May 1981; Heesterbeek and Roberts 2015). Indeed, ${\mathscr {R}}_0$, which characterizes the overall strength of an epidemic, is tied to the epidemic speed, *r*, which is the initial exponential growth rate of the epidemic (Dushoff and Park 1947). For the SIR model (Eq. (1)), we can derive *r* by assuming $S\approx N$ for small *t* and solving $dI/dt=(\beta -\gamma )I$ so that $I(t) = \exp ((\beta -\gamma )t)$ and $r=\beta -\gamma =\gamma ({\mathscr {R}}_0-1)$. (Note that the exact formula connecting ${\mathscr {R}}_0$ and *r* depends on the specification of the model, so other models may have different relationships between ${\mathscr {R}}_0$ and *r*). The threshold of ${\mathscr {R}}_0>1$ can be framed instead as $r>0$, which is convenient in some contexts, particularly in the exponential growth phase of an epidemic or when investigating interventions that target individuals (Diekmann et al. 2010; Dushoff and Park 1947).

The approximations that we made to think through the intuition for ${\mathscr {R}}_0$ of the SIR model—namely $I\approx 1$ and $S\approx N$—will not be true over the course of the outbreak, so ${\mathscr {R}}_0$ is really only defined around the idea of a potential outbreak. But, we may be interested in what the expected number of secondary cases is in the midst of a real outbreak. The *effective reproduction number*, ${\mathscr {R}}(t)={\mathscr {R}}_0S(t)/N$, captures this concept. Why this form? Recall that in the intuitive derivation of ${\mathscr {R}}_0$ we said an infectious person infects $\beta S/N$ people per day. For ${\mathscr {R}}_0$, we assumed $S\approx N$, but that approximation is no longer valid in middle of the outbreak, and the current fraction of susceptibles *S*(*t*)/*N* needs to be accounted for. When working with infectious disease interventions, we are often trying to get ${\mathscr {R}}(t)<1$, so that the epidemic will die out. For example, we may want to introduce a supplemental vaccination campaign with the goal of moving people from the susceptible compartment to the immune compartment, effectively shorting out potential transmission chains. In the COVID-19 pandemic, there was a lot of discussion about “flattening the curve” (Boumans 2021). Essentially, the goal was to use social distancing to lower ${\mathscr {R}}(t)$ to get a lower, wider outbreak instead of a shorter, faster outbreak. While this outcome would likely reduce the cumulative incidence somewhat, the primary goal was to keep the number of infections at any given time low enough that they would not overwhelm healthcare systems.

The reproduction number’s threshold property (such that an epidemic will die out if ${\mathscr {R}}_0$ or ${\mathscr {R}}(t)<1$) is closely tied to the concept of *herd protection* (Fine 1993). We don’t need everyone in the population to be immune to effectively interrupt infection; we just need enough people to be immune that a single person will infect fewer than one susceptible person on average. Because not everyone can be immunized for logistical or medical reasons, we often rely on herd protection to keep these unvaccinated people safe. The fraction of the population that need to be vaccinated to achieve herd protection, denoted *H*, differs from disease to disease based on the reproduction number,

2 $$\begin{aligned} H= 1-\frac{1}{{\mathscr {R}}_0}. \end{aligned}$$

Remember that ${\mathscr {R}}_0$ is defined for a fully susceptible population, where $S(0)/N\approx 1$. Here, we use the effective reproduction number at the start of the epidemic, accounting for the vaccinated fraction with $S(0)/N= 1-H$, so that ${\mathscr {R}}(0)={\mathscr {R}}_0(S(0)/N)={\mathscr {R}}_0(1-H)$. Solving this equation when ${\mathscr {R}}(0)=1$ results in the above expression for *H*. Given this relationship, we will only need relatively little vaccination coverage to control some diseases (around 50% for some strains of seasonal influenza) but will need to vaccinate almost everyone for very infectious disease, like measles (which requires upwards of 95% coverage). Vaccine hesitancy is a big concern to many people in public health, because diseases that were once virtually eradicated can regain a foothold when coverage dips too low, even locally. Population assortativity, which describes the situation where people are more likely to contact others with similar demographic characteristics (e.g., age, race, religious affiliation), can result in lower-than-expected levels of herd protection because the well-mixed assumptions that underlie the above equations are violated (Peeples 2019).

This concept of the basic reproduction number as a measure of infection control can be generalized into two related quantities, the type and target reproduction numbers (Roberts and Heesterbeek 2003; Shuai et al. 2013). Instead of asking what fraction of the at-risk population as a whole needs to be protected (e.g., through vaccination) to eliminate the disease, we can ask about targeting specific subgroups by calculating type reproduction numbers (e.g., targeting only young women for the human papillomavirus vaccine) or certain transmission pathways by calculating target reproduction numbers (e.g., the use of bed nets for reducing transmission to humans through mosquito feeding).

In practice, ${\mathscr {R}}_0$ can be calculated from data in a number of ways. For example, it can be estimated from the initial growth rate *r* of an epidemic, the cumulative incidence of infection after an epidemic, or age-specific prevalence of an endemic disease (Dietz 1993). Data-driven approaches for estimating ${\mathscr {R}}(t)$ are particularly attractive when transmission varies over time (Gostic et al. 2020). Alternatively, ${\mathscr {R}}_0$ can be calculated as a function of an infectious disease model’s parameters, as explored in the next sections. In this case, ${\mathscr {R}}_0$ can be calculated from known or assumed values of parameters in addition to values determined through parameter estimation (curve fitting). Indeed, there is a rich literature on parameter identifiability, that is, knowing whether parameters can be determined uniquely from available data, such as epidemic curves (Tuncer and Le 2018; Eisenberg et al. 2013; Kao and Eisenberg 2018; Dankwa et al. 2022; Massonis et al. 2021; Brouwer et al. 2018). Once we have these values, how do we develop a formula for ${\mathscr {R}}_0$ as a function of these parameters?

## The Next Generation Method

Several methods exist for calculating the ${\mathscr {R}}_0$ of an infectious disease epidemic model as a function of its parameters (Heffernan et al. 2005). In addition to helping to estimate the value of ${\mathscr {R}}_0$, knowing how ${\mathscr {R}}_0$ is a function of the model parameters aids in understanding the disease system and can help to determine disease control strategies. One of the most rigorous and commonly used approaches to calculate ${\mathscr {R}}_0$ as function of the model parameters is the next generation method. This method was developed by Diekmann, Heesterbeek, and colleagues (Diekmann et al. 1990; Diekmann and Heesterbeek 2000; Diekmann et al. 2010) and further explored by van den Driessche and Watmough (Van Den Driessche and Watmough 2002), among others, and we refer the reader to these references for the more technical details. The first step in this method is to distinguish between states in the model that represent infected individuals and those that do not, as well as between terms in the equations that represent new infections and those that do not. In the SIR model (Eq. (1)), only state *I* is infected, and the term $\beta (S/N)I$ represents new infections while the term $\gamma I$, which represents recoveries, does not. For a system of ordinary differential equations representing an infectious disease model, denote the vector of infected states by *x* and the vector of uninfected states by *y*. Denote a point in the state space, that is, a partition of the population *N* into each compartment, by (*x*, *y*). Then, the disease-free equilibrium is the point in state space where all individuals are susceptible, denoted $(0,y_0)$. For the SIR model, $x=I$, $y=(S,R)$, and $y_0=(N,0)$. For each infected compartment *i*, let ${\mathscr {F}}_i(x,y)$ be the rate at which previously uninfected people enter compartment *i*. Let ${\mathscr {V}}_i(x,y)$ be the rate of transfer of individuals out of compartment *i* minus the rate of transfer into compartment *i*. Then

3 $$\begin{aligned} \frac{dx_i}{dt} ={\mathscr {F}}_i(x,y)-{\mathscr {V}}_i(x,y). \end{aligned}$$

Let *F* and *V* be the Jacobian matrix (the matrix of partial derivatives) of ${\mathscr {F}}$ and ${\mathscr {V}}$, respectively, evaluated at the disease-free equilibrium (DFE), i.e., the matrices whose entries are

4 $$\begin{aligned} \begin{aligned} F_{ij}= \frac{\partial {\mathscr {F}}_i}{\partial {x_j}}(0,y_0), \quad V_{ij}= \frac{\partial {\mathscr {V}}_i}{\partial {x_j}}(0,y_0). \end{aligned} \end{aligned}$$

The matrices *F* and *V* can be used to succinctly write the equations for the infected compartments in the ODE linearized at the disease-free equilibrium,

5 $$\begin{aligned} \frac{dx}{dt} = (F-V)x \end{aligned}$$

Linearization—that is defining a new, linear ODE from the linear terms of the Taylor expansion of a nonlinear ODE, typically around an equilibrium point—is often used to determine the stability an equilibrium point of a nonlinear ODE. If a nonlinear ODE is linearized around an equilibrium point, the stability of that equilibrium point in the linearized ODE, which is straightforward to calculate, is the same as the stability of that equilibrium point in the original, nonlinear ODE. We will revisit this linearized system in Sect. 4. The SIR model has a single infected compartment, and $F=\beta$ and $V=\gamma$. We previously saw the linearized SIR model, $dI/dt=(\beta -\gamma )I$, when calculating the epidemic speed in the previous section. Additional examples will be explored in Sect. 5.

The matrix $K=FV^{-1}$ is called the next generation matrix. Why this form, and how does one interpret the entries of this matrix? In the case that ${\mathscr {V}}$ represents individuals changing states (we will complicate and generalize this situation in some later examples), the authors van den Driessche and Watmough give us a succinct answer (Van Den Driessche and Watmough 2002):

To interpret the entries of $FV^{-1}$ and develop a meaningful definition of $R_0$, consider the fate of an infected individual introduced into compartment *k* of a disease-free population. The (*j*, *k*) entry of $V^{-1}$ is the average length of time this individual spends in compartment *j* during its lifetime, assuming that the population remains near the DFE and barring reinfection. The (*i*, *j*) entry of *F* is the rate at which infected individuals in compartment *j* produce new infections in compartment *i*. Hence, the (*i*, *k*) entry of the product $FV^{-1}$ is the expected number of new infections in compartment *i* produced by the infected individual originally introduced into compartment *k*.

The basic reproduction number is defined to be the spectral radius—that is, the magnitude of the largest eigenvalue—of the matrix $K=FV^{-1}$, denoted $\rho (K)$. The eigenvalues $\lambda$ of *K* can be found by solving the characteristic equation $\text {det}(\lambda I- K)=0$, where *I* is the identity matrix the same size as *K*. An important feature of *K* is that all entries are nonnegative; this implies (through extensions of the Perron–Frobenius Theorem (Meyer 2000)) that *K*’s largest eigenvalue is nonnegative (and thus equal to $\rho (K)$) and is associated with a nonnegative eigenvector $\nu$. These properties are important, as we will need $\rho (K)$ and $\nu$ to have realistic interpretations in terms of populations dynamics. If *K* is irreducible, that is, if every type of infection can cause an infection of every other type (possibly via an intermediate infection type), then there are no nonnegative eigenvectors other than $\nu$. (If *K* is reducible, then there may be degenerate initial conditions).

The next generation theorem tells us that our continuous ODE system is stable (no outbreak) if and only if $\rho (K)<1$ and is unstable (outbreak) if and only if $\rho (K)>1$ (Diekmann et al. 1990; Diekmann and Heesterbeek 2000; Diekmann et al. 2010; Van Den Driessche and Watmough 2002). Why does the spectral radius of the next generation matrix control the behavior of the infectious disease system? The answer may not be immediately intuitive.

## The Basic Reproduction Number as a Spectral Radius

### The Next Generation Matrix as a Linear Transformation

Let $z_0=(z_{0,1},\dots ,z_{0,m})$ be a vector denoting *M* individuals across *m* infected compartments at the start of an epidemic, i.e., $z_0$ are the patient zeros of the outbreak. Then, we can apply the next generation matrix as a linear transformation $K:{\mathbb {R}}^{m,+}\rightarrow {\mathbb {R}}^{m,+}$ on this vector $z_0$ of infected individuals by matrix multiplication (taking $z_0$ as a column vector). The result, $z_1=Kz_0$ is the vector of individuals in each of the *m* infected compartments in the *next* generation, i.e., all of the individuals that were infected by the individuals in $z_0$. Consider the following next generation matrix *A* for a disease system with two infectious compartments, say adults and children, respectively,

6 $$\begin{aligned} A=\begin{bmatrix} 1.58 &{} 0.84\\ 0.14 &{} 1.72 \end{bmatrix}. \end{aligned}$$

One average, one adult will infect 1.58 adults and 0.14 children, and one child will infect 0.84 adults and 1.72 children. If we start with one individual of each type, $z_0=(1,1),$ then we expect the next generation to be $z_1=Az_0=(2.42, 1.86)$. Two individuals (one of each type) initially infected are expected to infect 4.28 people. Subsequent generation sizes and distributions can be calculated with further applications of *A*, i.e., the *n*th generation is $z_n=A^nz_0$.

While it makes intuitive sense that we should expect an outbreak when the next generation of infected individuals is larger than the previous generation, it may be less obvious how this discrete process corresponds to our original continuous ODE model. To understand the connection, it is important to first remember that our goal is not to find an alternate, discrete approach to simulating the disease system. Instead, our goal is to determine the threshold that controls the stability of the ODE system near the disease-free equilibrium, that is, find the threshold that determines whether or not there will be an outbreak. To that end, let’s consider the linearized ODE disease system near the disease free-equilibrium. As we saw in Sect. 3, this linearized system can be written as

7 $$\begin{aligned} \frac{d}{dt}x(t) = (F-V)x(t), \end{aligned}$$

where *x*(*t*) is the vector of the number of people in each infected compartment over time.

Now, let’s distinguish between each generation in our ODE model, beginning with the initial generation $x_0(t)$ starting on the initial condition of the system, $x_0(0)$. (Note: $x_0$ cannot begin exactly on the disease-free equilibrium or there would be no disease in the system, but we are considering a value $x_0(0)$ near the disease-free equilibrium). Let $x_n(t)$ be the vector of infectious individuals in each infected class at time *t* who were part of the *n*th generation. Then, *x*(*t*), the vector of the number of individuals in each of the infected compartments over time, regardless of generation, is the sum over all of the generations, $x(t) = \sum _{n=0}^\infty x_n(t)$. The following infinite-dimensional ODE system describes how the number of people in each infected compartment in each generation change over time (Hurford et al. 2010).

8 $$\begin{aligned} \begin{aligned} \frac{dx_0}{dt}&= - V x_0,\\ \frac{dx_1}{dt}&= F x_0 - V x_1,\\ \frac{dx_2}{dt}&= F x_1 - V x_2,\\&\vdots \\ \frac{dx_n}{dt}&= F x_{n-1} - V x_n,\\&\vdots \\ \end{aligned} \end{aligned}$$

The vector $z_n$ (as in the discrete system above) is the total number of new infections of each type in generation *n*, irrespective of when they were infected. It is calculated as the cumulative number of people in the *n*th generation entering each infected class, namely $z_n=\int _0^\infty F x_{n-1}(t)\,dt$. (Define $z_0=x_0(0)$). A recursive relationship can be derived for $z_n$ from the infinite dimensional ODE system above (Hurford et al. 2010), namely $z_n=FV^{-1}z_{n-1}$. Hence, the discrete process of applying the next generation matrix exactly describes how the size of each generation changes in our linearized ODE model. In fact, the next generation theorem tells us that the stability of the discrete process of applying the next generation method is the same as that of the linearized ODE (and thus the original ODE) (Van Den Driessche and Watmough 2002; Hurford et al. 2010).

It is important to emphasize that this discussion is in the context of the linearized ODE system near the disease-free equilibrium. Because nonlinear ODE epidemic models quickly leave this regime, we should not expect the behavior of those trajectories to match these iterations of the next generation matrix. Hence, applying the next generation matrix *K* is not a substitute for the ODE system; instead the next generation matrix should be seen as an interpretable object that tells us whether we expect an outbreak for a given disease system.

Although we now understand how using the next generation matrix as a linear process relates to the dynamics of our disease system, we still need to answer the question of why the spectral radius of *K* is the key threshold for the stability of these epidemic models.

### The Geometry of the Next Generation Matrix

Recall from Sect. 3 that the spectral radius of *K* is the magnitude of the largest eigenvalue of *K*. To understand why the spectral radius is important, let us consider a geometric interpretation of an eigenvalue $\lambda$ and corresponding eigenvector $\nu$ of the next generation matrix *K*. Recall that an eigenvalue $\lambda$ and eigenvector $\nu$ are a scalar and vector, respectively, such that

9 $$\begin{aligned} K\nu = \lambda \nu . \end{aligned}$$

So, $\nu$ represents a special distribution of infected individuals such that new generations will maintain the same relative distribution among the infected classes. For the next generation matrix *A* defined in Eq. (6), $\nu =(2,1)$ is an eigenvector because $A\nu =2\nu$. Starting with 2 infected adults and 1 infected child will result in 4 infected adults and 2 infected children. The proportion of individuals in each infected compartment stayed the same in the next generation, but the overall size doubled.

Why are these eigenvalues and eigenvectors important? Let’s take a step back and consider the space of all possible initial generations $z_0=(z_{0,1},\dots ,z_{0,m})$ with a fixed total population *M*, that is, all distributions of *M* individuals into each of the *m* infected compartments. What we are describing here is a circle (or, more generally, an *m*-sphere) with radius *M*, i.e., the set of all vectors $z_0\in {\mathbb {R}}^{m,+}$ with norm *M*. Although most of us are most familiar with the ${\mathscr {L}}^2$ (Euclidean) norm that governs distance in the usual sense, with $||z_0||_2=\sqrt{\sum _i z_{0,i}^2}$, the ${\mathscr {L}}^1$ norm (sometimes called the taxicab or absolute value norm), with $||z_0||_1=\sum _i |z_{0,i}|$, is more natural for considering a partition of a fixed population into different compartments. For example, it makes more biological sense to say that a vector (3, 4) denoting 3 latently infected and 4 infectious people has size $3+4=7$ (${\mathscr {L}}^1$) rather than $\sqrt{3^2+4^2}=5$ (${\mathscr {L}}^2$). What works well for distance does not necessarily make sense for populations. Interestingly, our “circle" of radius *M* has a diamond shape in ${\mathscr {L}}^1$ norm. To see this, consider the set of vectors in ${\mathbb {R}}^{2,+}$ with norm *M*. In the ${\mathscr {L}}^2$ norm, this looks like the arc of a circle, with $z_{0,2}=\sqrt{M^2-z_{0,1}^2}$, but in the ${\mathscr {L}}^1$ norm, we have the linear relationship $z_{0,2} = M- z_{0,1}$.

**Fig. 1:**
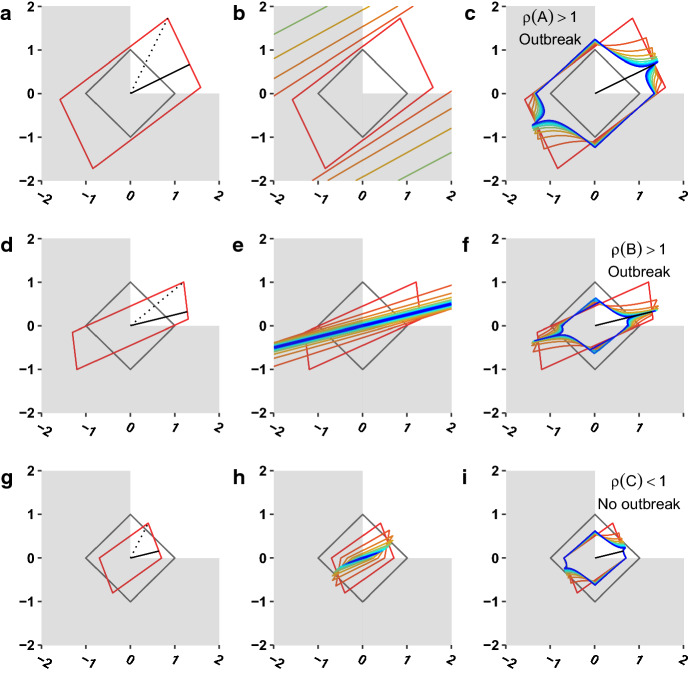
**a** $\{Az_0: z_0\in {\mathbb {R}}^2, |z_0|=1\}$. The solid black line segment denotes the eigenvector of the dominant eigenvalue of *A*, while the dotted black line segment is the largest generation of $\{Az_0\}$, corresponding to |*A*|. **b** $\{A^nz_0: z_0\in {\mathbb {R}}^2, |z_0|=1\}$. Each set of colored lines red, orange, etc., corresponds to $n=1,2,\dots$. c) $\{\frac{A^nz_0}{|A^nz_0|}\cdot \root n \of {|A^nz_0|}: |z_0|=1\}$. This set is the vectors in (**b**) scaled to the geometric mean norm over the *n* iterates of *A*. The solid black line segment denotes the eigenvector of the dominant eigenvalue of *A*. The colored lines correspond to values of *a*, as in (**b**). Plots (**d**), **e**, and **f** and (**g–i**) are analogous to (**a–c**) for matrices *B* and *C*. In each subfigure, the first quadrant is emphasized because it corresponds to realistic interpretations of population dynamics. (Colour figure online)

Now, we have the set of all possible initial generation vectors $z_0$ denoting *M* individuals partitioned into *m* infected compartments, $Z_0=\{z_0\in {\mathbb {R}}^{m,+}: |z_0|=M\}$. (We will use absolute value notation to denote the ${\mathscr {L}}^1$ norm, $||\cdot ||_1=|\cdot |$). Applying a next generation matrix *K* by matrix multiplication to a vector $z_0\in Z_0$ results in a new vector $z_1=Kz_0$, where $z_1$ represents the number of new infections of each type in the next generation. If we apply *K* to every vector in $Z_0$, i.e., every initial generation, we trace out a new shape in ${\mathbb {R}}^{m,+}$(Fig. 1a). Although the size (norm) of every vector $z_0$ was *M*, the size of each $z_1$ can be different. For example, for our next generation matrix *A* given in Eq. (6), the size of the next generation is $|(1.58, 0.14)|= 1.72$ for initial generation (1, 0) and is $|(0.84, 1.72)| = 2.56$ for initial generation (0, 1). We say that *K* stretches $z_0$, and the magnitude of this stretch is given by $|Kz_0|/|z_0|$. This stretch depends only on the relative sizes of the infected classes in $z_0$, e.g., matrix *A* stretches initial generations (1, 1) and (2, 2) by the same amount, $4.28/2=8.48/4=2.14$. The largest possible stretch by *K* is called the operator norm of *K* and is denoted $|K|=\max _{|z_0|=1} |Kz_0|$, which we will use below. Here, we see that each generation produces a next generation of a different size depending on the distribution of individuals among the infected compartments. But, then, how do I know what the expected generation size is? And what does this have to do with the spectral radius?

To answer these questions, let’s first think about what happens when we apply matrix *K* to the set of initial generation vectors *n* times. This process calculates the size of the *n*th generation of infected people and the distribution of the individuals among the *m* infected classes. The original shape becomes more exaggerated as the size of the subsequent generations increases. Iterations of this process tell us about the long-term behavior of the system linearized at the disease-free equilibrium for each possible initial condition (Fig. 1b). However, we are interested not in the long-term values, per se, but in the expected number of new infections per generation, averaged over these multiple generations. Because $\rho (K)$ is the largest positive eigenvalue of *K*, the relative distribution of individuals across the *m* classes gets closer and closer to that of the eigenvector $\nu$ that corresponds to $\rho (K)$ as we calculate successive generations, i.e., iterations of the next generation matrix *K*. The influence of the other eigenvalues dies out quickly, so whatever the distribution of infection types is initially, the distribution of new infections converges to that of this eigenvector $\nu$.

How many new infections are in a new generation when we are near this stable distribution represented by $\nu$? That is our key quantity. To calculate this, we can scale our transformations so that magnitude of the stretch of the initial generation $z_0$ is the geometric mean stretch, that is $\root n \of {|K^nz_0|/|z_0|}$ as *n* increases. For example, recall next generation matrix *A* in Eq. (6), and consider $z_0=(1,1)$, $Az_0=(2.42,1.86)$, and $A^2z_0=(5.39,3.54)$. The stretch from the initial to the first generation was 2.14, and the stretch from the first to second generation was 2.09. The geometric mean stretch across these first two generations was $\sqrt{|(5.39,3.54)|/|(1,1)|}=2.11$. As we do this geometric scaling, the magnitude of stretch converges to $\rho (K)$ (Fig. 1c), per Gelfand’s Formula (Kozyakin 2009), using the operator norm notation defined above,

10 $$\begin{aligned} \rho (K)=\lim _{n\rightarrow \infty }\root n \of {|K^n|}. \end{aligned}$$

Hence the spectral radius gives the asymptotic average (i.e., asymptotic geometric mean) next generation size. In other words, for almost every initial generation, the average next generation has distribution $\nu$ and magnitude $\rho (K)$. We can intuitively think of this geometric interpretation of *K* as telling us that the first couple of generations of a new epidemic will quickly converge to a specific pattern, with the size of each generation increasing (or decreasing) by a factor of $\rho (K)$ and the distribution among infected compartments given by $\nu$. Now, we can at last understand why the spectral radius of *K* gives the expected size of an average next generation and why this basic reproduction number determines the stability of our ODE disease system near the disease-free equilibrium.

### Visualization with Example Next Generation Matrices

Let’s visualize the geometric interpretation of the next generation matrix in a few concrete examples. First, let’s revisit the next generation matrix *A* given in Eq. (6). Because *A*, is a nonnegative matrix, *A* has a largest real eigenvalue equal to the spectral radius and a corresponding nonnegative eigenvector. Matrix *A* has eigenvalues 2 and 1.3 and corresponding eigenvectors (2, 1) and $(3,-1)$. As we apply *A* to subsequent generations of infected individuals, these generations will converge toward a stable distribution of adults and children infected in each next generation. By normalizing the eigenvector corresponding to the largest eigenvalue, we can see that the distribution of new infections converges to 2/3 adults and 1/3 children. Regardless of initial distribution, the system moves to a stable distribution of new infections of both types, with an average of 2 new infections per infected individual overall.

In Fig. 1a, we plot the transformation $\{Az_0: |z_0|=1\}$ with the dominant eigenvector of *A*. Note that although we show the transformation on the full unit circle, we are primarily interested in the behavior in the first quadrant, ${\mathbb {R}}^{2,+}$ for biological realism. In Fig. 1b, we plot the set of transformations $\{A^nz_0:|z_0|=1\}$. The successive transformations become more exaggerated and rotate slightly. In Fig. 1c, we plot the set of points $\left\{ \frac{A^nz_0}{|A^nz_0|}\cdot \root n \of {|A^nz_0|}: |z_0|=1\right\}$. The norm of these transformations is $\root n \of {|A^n|}$, and we see that the direction and magnitude of the largest stretch of this increasingly long-term average behavior, converges toward the eigenvector $\nu$ and dominant eigenvalue $\rho (A)$ of the original transformation. Indeed, if we were to plot a uniform distribution of points on the original unit circle, the density of those transformed points in Fig. 1c would not be uniform but rather would be densest near the eigenvector. Whatever the initial conditions, the (linearized) outbreak dynamics converge to a particular magnitude and distribution. In this example, since $\rho (A)>1$, there will be an outbreak.

We repeat this exercise with two more matrices:

11 $$\begin{aligned} B&=\begin{bmatrix}1.30 &{} 1.20\\ 0.15 &{} 1.00 \end{bmatrix},\end{aligned}$$

12 $$\begin{aligned} C&=\begin{bmatrix} 0.70 &{} 0.40\\ 0 &{} 0.80 \end{bmatrix}. \end{aligned}$$

Matrix *B* has eigenvalues 1.6 and 0.7, and we expect an outbreak. Matrix *C*, has eigenvalues 0.8 and 0.7, so, despite the fact that infections of type 2 produce an average of $0.4+0.8=1.2>1$ infections in the next generation, we do not expect an outbreak. Note that *C* is not irreducible because infections of type 1 cannot make infections of type 2, which is why *C* can have two nonnegative eigenvectors. But, one of these eigenvectors corresponds to a degenerate initial condition (all type 1). All other initial conditions converge to the eigenvector associated with $\rho (C)$. Fig. 1d–i is analogous to Fig. 1a–c for matrices *B* and *C*.

## Symbolic Interpretation of the Basic Reproduction Number

One challenge in working with basic reproduction numbers calculated from the next generation method—particularly when using symbolic calculators—is interpreting and developing intuition for the form of ${\mathscr {R}}_0$ as a function of the model parameters. Accordingly, it is often easier to develop intuition for your model’s basic reproduction number if you calculate the next generation matrix by hand, grouping interpretable terms together. It is also often advisable to calculate ${\mathscr {R}}_0$ for one or more simplified models to get a better sense of how different parts of the model contribute to the form ${\mathscr {R}}_0$ for more complex models.

**Fig. 2:**
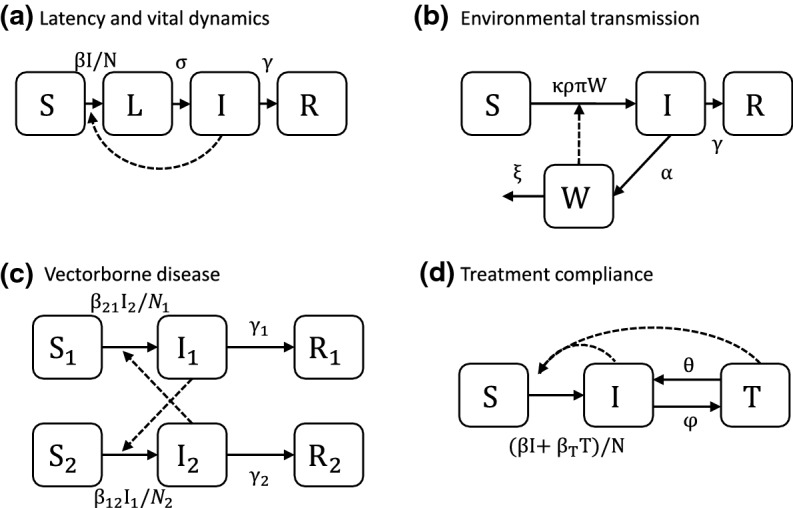
Compartmental Model Diagrams

Here, we explore the calculation and interpretation of ${\mathscr {R}}_0$ in four classic models (adapted in part from (Van Den Driessche and Watmough 2002; Li et al. 2009)): a model with latency and vital dynamics (Fig. 2a), a model with indirect transmission through the environment (Fig. 2b), a model of vectorborne disease (Fig. 2c), and a treatment-compliance model (Fig. 2d). These examples highlight a number of common forms that arise in ${\mathscr {R}}_0$ and which may help you develop intuition for interpreting the form of the basic reproduction number in your own models.

### A Model with Latency and Vital Dynamics

One simple extension of the SIR model is the SLIR model, in which we add a compartment *L*, which tracks the number of people with latent infections, who are not yet infectious (*L* is sometimes called the “exposed” compartment). A second extension is to include vital dynamics, i.e., birth and death, here represented by the parameter $\mu$.

13 $$\begin{aligned} \begin{aligned} \frac{dS}{dt}&= \mu N-\beta SI/N-\mu S,\\ \frac{dL}{dt}&=\beta SI/N -\sigma L-\mu L,\\ \frac{dI}{dt}&= \sigma L -\gamma I -\mu I,\\ \frac{dR}{dt}&= \gamma I- \mu R. \end{aligned} \end{aligned}$$

Our parameters have the following interpretations: $\mu$ is the birth/death rate, $\beta$ is the transmission rate, $1/\sigma$ is average time in the latent compartment, and $1/\gamma$ is the average time spent in the infectious compartment. Then there are two infected compartments, *L* and *I*. We write

14 $$\begin{aligned} {\mathscr {F}}&= \begin{bmatrix} \beta SI/N \\ 0\end{bmatrix}, \end{aligned}$$

15 $$\begin{aligned} {\mathscr {V}}&=\begin{bmatrix} (\sigma +\mu ) L \\ (\gamma +\mu )I-\sigma L\end{bmatrix}. \end{aligned}$$

Following Eq. (4), we calculate the matrix of partial derivatives of *F* and *V* with respect to *L* and *I* and evaluate them at the disease-free equilibrium, where $S=N$ and $L=I=R=0$.

16 $$\begin{aligned} F&= \begin{bmatrix} \frac{\partial }{\partial L}(\beta SI/N) &{} \frac{\partial }{\partial I}(\beta SI/N) \\ \frac{\partial }{\partial L}0 &{} \frac{\partial }{\partial I}0\end{bmatrix}_{S=N,L=I=R=0}=\begin{bmatrix} 0 &{} \beta \\ 0 &{} 0\end{bmatrix}, \end{aligned}$$

17 $$\begin{aligned} V&=\begin{bmatrix} \frac{\partial }{\partial L}( (\sigma +\mu ) L ) &{} \frac{\partial }{\partial I}( (\sigma +\mu ) L ) \\ \frac{\partial }{\partial L}((\gamma +\mu )I-\sigma L) &{} \frac{\partial }{\partial I}((\gamma +\mu )I-\sigma L)\end{bmatrix}_{S=N,L=I=R=0}\nonumber \\&=\begin{bmatrix} \sigma +\mu &{} 0 \\ -\sigma &{} \gamma +\mu \end{bmatrix}. \end{aligned}$$

Note that the *i*th component of the diagonal of *V* is rate of leaving compartment *i* (the sum of exponential rates gives the rate of the first event, whichever that ends up being). The other elements in the corresponding column are the rates of movement to the other infected compartments. The sum of a column gives the rate of becoming not infected (whether through recovery or death). Then,

18 $$\begin{aligned} V^{-1}=\begin{bmatrix}\frac{1}{\sigma +\mu } &{} 0 \\ \frac{\sigma }{(\sigma +\mu )(\gamma +\mu )} &{}\frac{1}{\gamma +\mu } \end{bmatrix}, \end{aligned}$$

and

19 $$\begin{aligned} FV^{-1} = \begin{bmatrix} \frac{\beta \sigma }{(\sigma +\mu )(\gamma +\mu )} &{} \frac{\beta }{\gamma +\mu }\\ 0 &{} 0 \end{bmatrix}. \end{aligned}$$

Why is the second row of the next generation matrix all zeros? Well, this row corresponds to the number of new people in compartment *I* given a person in either *L* or *I* near the disease-free equilibrium. Any new infections produce latent people, not infectious people directly.

The next generation matrix has two eigenvalues, 0 and

20 $$\begin{aligned} {\mathscr {R}}_0=\rho (FV^{-1}) = \left( \frac{\sigma }{\sigma +\mu }\right) \left( \frac{\beta }{\gamma +\mu }\right) . \end{aligned}$$

We see that the basic reproduction number of the SLIR model is the basic reproduction number of the SIR model (when including vital dynamics)—$\beta /(\gamma +\mu )$—times the fraction of exposed individuals who go on to become infectious—$\sigma /(\sigma +\mu )$. We also see that as $\sigma \rightarrow \infty$, that is, as transition out of the exposed compartment becomes instantaneous, the basic reproduction number converges to that of the SIR model, as we would expect. The basic reproduction number of this model accounts for a reduction in transmission in the system when a latent person dies before they can become infectious.

### A Model with Indirect, Environmental Transmission

When modeling many diseases, we often consider direct, person-to-person transmission, captured by the familiar $\beta$ term. However, with the exception of sexually transmitted infections, pathogen transmission is actually mediated by the environment and may be affected by environmental processes. For many diseases, the environmental dynamics are fast enough that the direct transmission approximation works well. However, when pathogens are persistent in the environment, the direct transmission approximation may no longer be valid, and we may want to explicitly model the concentration of pathogens in the environment. The basic reproduction number of this class of models will have a very different form from what we’ve seen for models with direct transmission.

Consider an infectious disease system with the familiar *S*, *I*, and *R* compartments. We additionally track the concentration of pathogens in the water system *W*. In this model, people become infectious not through contact with one another but by drinking the water. People drink $\rho$ volume of water $\kappa$ times a day and each pathogen has a probability $\pi$ of causing infection (see (Brouwer et al. 2017) for a discussion of more advanced dose–response functions). Some models parameterize $\beta _W=\kappa \rho \pi$, although this $\beta _W$ is subtly different from the $\beta$ of the SIR model: while the $\beta$ of the SIR model is often parameterized from the perspective of the infectious person (i.e., how fast are infectious people transmitting to susceptible people), $\beta _W$ is from the perspective of the susceptible person (i.e., how fast are susceptible people getting infected from the water). Finally, infectious people shed pathogens into the water at rate $\alpha$, and pathogens die-off in the environment at rate $\xi$. This model is a simple example of an SIWR-type model (Li et al. 2009; Tien and Earn 2010).

21 $$\begin{aligned} \begin{aligned} \frac{dS}{dt}&= -\kappa \rho \pi WS,\\ \frac{dI}{dt}&=\kappa \rho \pi WS-\gamma I,\\ \frac{dW}{dt}&= \alpha I - \xi W,\\ \frac{dR}{dt}&= \gamma I. \end{aligned} \end{aligned}$$

There are again two infected compartments—although the environment is not a class of infected individuals, it is “infected” by infected individuals and can infect susceptible individuals. However, since we want to calculate the basic reproduction number from the perspective of new *people* getting infected, we do not treat pathogens shedding into the water ($\alpha I$) as creating new infections.

We write

22 $$\begin{aligned} {\mathscr {F}}&= \begin{bmatrix} \kappa \rho \pi WS \\ 0\end{bmatrix}, \end{aligned}$$

23 $$\begin{aligned} {\mathscr {V}}&=\begin{bmatrix} \gamma I \\ \xi W- \alpha I\end{bmatrix}, \end{aligned}$$

and, as before, we calculate *F* and *V* at the matrix of partial derivatives evaluated at the disease-free equilibrium, where $S=N$ and $I=W=R=0$.

24 $$\begin{aligned} F&= \begin{bmatrix} 0 &{} \kappa \rho \pi N\\ 0 &{} 0\end{bmatrix} ,\end{aligned}$$

25 $$\begin{aligned} V&=\begin{bmatrix}\gamma &{} 0 \\ -\alpha &{} \xi \end{bmatrix}. \end{aligned}$$

Then

26 $$\begin{aligned} V^{-1}=\begin{bmatrix}\frac{1}{\gamma } &{} 0 \\ \frac{\alpha }{\gamma \xi } &{} \frac{1}{\xi }\end{bmatrix}. \end{aligned}$$

It is worth discussing the lower left entry of $V^{-1}$ specifically. In our original discussion, we defined this entry as the average length of time that an individual in *I* would spend in *W*. However, because $\alpha$ does not exactly represent compartment transfer, the original interpretation that van den Driessche & Watmough gave us in Sect. 2 does not quite make sense here. We have to complicate and generalize the interpretation. This entry indicates that an individual in *I* is responsible for an average $\alpha /\gamma$ pathogens living for an average $1/\xi$ time.

In this example, the calculation of $K=FV^{-1}$ and $\rho (K)$ is straightforward:

27 $$\begin{aligned} {\mathscr {R}}_0 = \frac{\alpha \kappa \rho \pi N}{\gamma \xi }. \end{aligned}$$

How do we interpret this parameter combination? First, an infectious person will shed $\alpha$ pathogens per unit volume into the environment per day, and thus an average of $\alpha /\gamma$ pathogens over their infectious lifetime. Each pathogen can infect new people for $1/\xi$ days (here, we neglect pathogen loss from the environment due to ingestion for simplicity, but some models track it explicitly (Li et al. 2009)). If $\kappa \rho N$ is the volume of the environment ingested per day (by the initially susceptible population) and $\pi$ is the per pathogen probability of infection, then $\kappa \rho \pi N$ is the per-pathogen number of new infections per day. Putting this all together, we arrive at the average number of people that a single infectious person will infect over their infectious lifetime. This example highlights that the basic reproduction number can be calculated and interpreted for models with indirect transmission but that we may have to move beyond the conception of ${\mathscr {R}}_0$ as the number of people that someone directly infects on average.

### A Model with Vector Transmission

Another class of models that do not use direct transmission is vectorborne disease models. One important classes of vectorborne diseases are arboviruses, which include dengue, chikungunya, yellow fever, and many others. Malaria is caused by a parasite spread by mosquitoes, and Lyme disease is caused by a bacteria spread by ticks. In vectorborne disease models, we have two or more classes of hosts that can each be susceptible, infectious, or recovered (although modeling recovered vectors is often not needed). Each class of host only transmits the disease to the other class and not directly to other members of their own class (e.g., mosquitoes infect humans but not other mosquitoes).

Here, we use a very simple model with two classes of individuals (1 and 2), each of which can be S, I, or R. Infectious members of each class transmit only to the other class with rates $\beta _{12}$ and $\beta _{21}$. Note that we have chosen to parameterize these rates from the point of view of the infectious individual, not the susceptible individual; this distinction means that we scale our rates by the population of the class that is being transmitted to, not the population that is transmitting. (Other choices are possible—for vectorborne models, it is common to specify the parameters so that you are dividing by the number of humans in both cases. Regardless of how you want to parameterize, it is important to be sure that the values used for the $\beta _{12}$ and $\beta _{21}$ parameters match their interpretation). Our equations are

28 $$\begin{aligned} \frac{dS_1}{dt}&= -\beta _{21} S_1I_2/N_1,\nonumber \\ \frac{dI_1}{dt}&= \beta _{21} S_1I_2/N_1 -\gamma _1 I_1,\nonumber \\ \frac{dR_1}{dt}&= \gamma I_1,\nonumber \\ \frac{dS_2}{dt}&= -\beta _{12} S_2I_1/N_2,\nonumber \\ \frac{dI_2}{dt}&= \beta _{12} S_2I_1/N_2 -\gamma _2 I_2, \frac{dR_2}{dt}= \gamma I_2. \end{aligned}$$

At this point, we face an important decision: do new infections in each class of individuals count as new infections? The answer depends on your interpretation of the model. If class 1 represents humans and class 2 represents mosquitoes, we probably only care about new infections in humans. Consider instead a model representing a sexually transmitted infection in a fully heterosexual population of men and women. (Note that this is not a realistic representation of real populations, given the spectra of sexuality and gender, the violation of the well-mixed contact assumption by the assortativity of sexual partners by age and other characteristics, and the violation of the assumption of homogeneity of contact rates—but it is nonetheless helpful to build intuition starting from the most simplified models). In this model, we would count new infections in both classes.

To start with, let’s continue with the vectorborne example, and write

29 $$\begin{aligned} {\mathscr {F}}= & {} \begin{bmatrix} \beta _{21}S_1I_2/N_1 \\ 0\end{bmatrix}, \end{aligned}$$

30 $$\begin{aligned} {\mathscr {V}}= & {} \begin{bmatrix} \gamma _1 I_1 \\ \gamma _2 I_2 - \beta _{12} S_2I_1/N_2 \end{bmatrix}. \end{aligned}$$

and evaluate the matrices of partial derivatives at the disease-free equilibrium, where $S_1=N_1$, $S_2=N_2$, and all other compartments are 0,

31 $$\begin{aligned} F= & {} \begin{bmatrix} 0 &{} \beta _{21} \\ 0 &{} 0\end{bmatrix} ,\end{aligned}$$

32 $$\begin{aligned} V= & {} \begin{bmatrix} \gamma _1 &{} 0 \\ -\beta _{12} &{} \gamma _2 \end{bmatrix}. \end{aligned}$$

Then

33 $$\begin{aligned} K= & {} \begin{bmatrix} 0 &{} \beta _{21} \\ 0 &{} 0\end{bmatrix} \begin{bmatrix} \frac{1}{\gamma _1} &{} 0 \\ \frac{\beta _{12}}{\gamma _1\gamma _2} &{} \frac{1}{\gamma _2} \end{bmatrix},\end{aligned}$$

34 $$\begin{aligned}= & {} \begin{bmatrix} \frac{\beta _{21}\beta _{12}}{\gamma _1\gamma _2} &{} \frac{\beta _{21}}{\gamma _2} \\ 0 &{} 0\end{bmatrix}. \end{aligned}$$

Note, again, that the lower left entry of $V^{-1}$ requires the generalized interpretation we discussed in the previous example. From *K*, we can see

35 $$\begin{aligned} {\mathscr {R}}_0 = \frac{\beta _{21}\beta _{12}}{\gamma _1\gamma _2}. \end{aligned}$$

The basic reproduction number in this interpretation is the number of human infections an average human infectious person makes over their infectious lifetime. We see that it is the average number of mosquito infections a human makes times the average number of human infections a mosquito makes.

But what if we care about new infections in both classes? We have to rewrite

36 $$\begin{aligned} {\mathscr {F}}&= \begin{bmatrix} \beta _{21}S_1I_2/N_1 \\ \beta _{12} S_2I_1/N_2\end{bmatrix}, \end{aligned}$$

37 $$\begin{aligned} {\mathscr {V}}&=\begin{bmatrix} \gamma _1 I_1 \\ \gamma _2 I_2 \end{bmatrix}, \end{aligned}$$

so that

38 $$\begin{aligned} K&= \begin{bmatrix} 0 &{} \beta _{21} \\ \beta _{12} &{} 0\end{bmatrix} \begin{bmatrix} \frac{1}{\gamma _1} &{} 0 \\ 0 &{} \frac{1}{\gamma _2} \end{bmatrix},\end{aligned}$$

39 $$\begin{aligned}&=\begin{bmatrix} 0 &{} \frac{\beta _{21}}{\gamma _2} \\ \frac{\beta _{12}}{\gamma _1} &{} 0\end{bmatrix}. \end{aligned}$$

The next generation matrix looks very different when using this interpretation. A little linear algebra calculation shows that

40 $$\begin{aligned} {\mathscr {R}}_0 = \sqrt{\frac{\beta _{21}\beta _{12}}{\gamma _1\gamma _2}}. \end{aligned}$$

Now, instead of the product of the average number of infections generated in the other class, we get the geometric mean. In the previous interpretation, transmission had to complete a 2-cycle (back and forth) to be considered to be infecting a new individual; in this interpretation, both directions are considered to be infecting a new individual, so we are taking an average over each transmission direction. Different definitions of “new infections” result in different reproduction numbers (Cushing and Diekmann 2016). More broadly, the square root is a common feature of models with two subpopulations (O’Regan et al. 2015; Fenton et al. 2015; Roberts and Heesterbeek 2018; Brouwer et al. 2015) because it arises from the solution to the quadratic characteristic polynomial of *K*. Indeed, the reader might like to explore how the formula for $R_0$ changes in the above model if we included both within- and between-group transmission.

Despite having different interpretations, both of the ${\mathscr {R}}_0$s we derived for this model have the mathematical property of controlling the stability of the disease-free equilibrium. If ${\mathscr {R}}_0$ in the first interpretation is greater than 1, then so too will ${\mathscr {R}}_0$ in the second interpretation. These models highlight how the basic reproduction number is both an important mathematical threshold and an epidemiological quantity that depends on our interpretation of the disease system.

Because there is not always a single valid interpretation, it is worth asking how we know whether a choice to include a term in ${\mathscr {F}}$ vs. in ${\mathscr {V}}$ is valid. A greater discussion of the assumptions underlying the next generation theorem is given in Van Den Driessche and Watmough (2002), but a decomposition is mathematically valid as long as the entries of *F* and $V^{-1}$ are all nonnegative and the eigenvalues of the matrix $-V$ have negative real parts (Hurford et al. 2010).

### A Treatment-Compliance Model

In this final example, we address a common challenge that arises in interpreting ${\mathscr {R}}_0$ in models with bidirectional movement between two infected compartments (i.e., individuals can go back and forth between compartments). This kind of model structure can arise, for example, if there are multiple disease stages through which one can progress and regress, e.g., asymptomatic and symptomatic stages of herpes simplex virus. This structure can also occur if infected people can switch between compartments as their characteristics change. A classic example of this kind of model is long-term disease treatment with lapses in treatment compliance, e.g., for HIV or tuberculosis treatment (Van Den Driessche and Watmough 2002).

For the purposes of illustration, we use a very simple model with two classes of infected people with bidirectional movement. Here, *I* represents untreated infected individuals, and *T* represents treated infected individuals. We can think of this as a simplified model of treatment compliance. Here, treatment might reduce infectiousness and reduce the mortality rate. Parameters $\beta$ and $\beta _T$ are contact rates times transmission probabilities for people not on treatment and on treatment, respectively, $\varphi$ is the rate of going on treatment, $\theta$ is the rate of treatment lapse, and $\nu$ and $\nu _T$ are the disease-related death rates for people not on treatment and on treatment, respectively.

41 $$\begin{aligned} \begin{aligned} \frac{dS}{dt}&= -S(\beta I +\beta _T T)/N\\ \frac{dI}{dt}&= S(\beta I +\beta _T T)/N -\varphi I + \theta T-\nu I\\ \frac{dT}{dt}&=\varphi I - \theta T -\nu _T T\\ \end{aligned} \end{aligned}$$

We have

42 $$\begin{aligned} {\mathscr {F}}&= \begin{bmatrix} S(\beta I +\beta _T T)/N \\ 0\end{bmatrix}, \end{aligned}$$

43 $$\begin{aligned} {\mathscr {V}}&=\begin{bmatrix} (\nu +\varphi ) I-\theta T \\ (\nu _T+\theta )T -\varphi I\end{bmatrix}, \end{aligned}$$

and evaluate the matrices of partial derivatives at the disease-free equilibrium, where $S=N$ and $I=T=0$,

44 $$\begin{aligned} F&= \begin{bmatrix} \beta &{} \beta _T \\ 0 &{} 0\end{bmatrix}, \end{aligned}$$

45 $$\begin{aligned} V&=\begin{bmatrix} \nu +\varphi &{} -\theta \\ -\varphi &{} \nu _T+\theta \end{bmatrix}. \end{aligned}$$

Then,

46 $$\begin{aligned} V^{-1}=\begin{bmatrix}\frac{ \nu _T+\theta }{(\nu +\varphi )( \nu _T+\theta )-\varphi \theta } &{} \frac{ \theta }{(\nu +\varphi )( \nu _T+\theta )-\varphi \theta } \\ \frac{ \varphi }{(\nu +\varphi )( \nu _T+\theta )-\varphi \theta } &{}\frac{\nu +\varphi }{(\nu +\varphi )( \nu _T+\theta )-\varphi \theta } \end{bmatrix}. \end{aligned}$$

Here, $V^{-1}$ is much harder to interpret than in the previous examples. The determinant of *V*, namely $(\nu _1+\varphi )( \nu _2+\theta )-\varphi \theta$, is more complicated and does not simplify with the other terms. Indeed, how are we to interpret this parameter combination?

Consider an individual with an untreated infection. They can either die with probability $\frac{\nu }{\nu +\varphi }$ or they can start treatment with probability $\frac{\varphi }{\nu +\varphi }$. (Incidentally, thinking in these terms—identifying jumping probabilities—is the first step to transitioning to a stochastic framework). Similarly, a person on treatment can die with probability $\frac{\nu _T}{\nu _T+\theta }$ or they can stop taking their treatment with probability $\frac{\theta }{\nu _T+\theta }$. The probability of starting in one compartment, jumping to the other, and then jumping back is

47 $$\begin{aligned} p:=\frac{\varphi \theta }{(\nu +\varphi )(\nu _T+\theta )}. \end{aligned}$$

The probability of jumping back and forth twice is $p^2$, and so on. Given that one starts out not on treatment, what is the expected number of times that one will not be on treatment? Let us count the times (Van Den Driessche and Watmough 2002). We start with one visit since one starts in that compartment. Then, we must add one for each additional visit, times the probability that the visit occurs. So, the expected number of visits is

48 $$\begin{aligned} 1+p+p^2+p^3+\dots =\frac{1}{1-p}. \end{aligned}$$

We can arrive at this number using some basic probability theory as well. Let *X*, a random number, be the number of visits to compartment *I*, and let *Z* be the event of a return visit to compartment *I*. Using the law of total expectation

49 $$\begin{aligned} \begin{aligned} E[X]&=1+P(Z)E[X|Z]+(1-P(Z))E[X|{\bar{Z}}]\\&=1+pE[X]+0 \end{aligned} \end{aligned}$$

Solving for *E*[*X*], we get

50 $$\begin{aligned} E[X]=\frac{1}{1-p}. \end{aligned}$$

In terms of our model, this expected number of visits is

51 $$\begin{aligned} \frac{1}{1-\frac{\varphi \theta }{(\nu +\varphi )(\nu _T+\theta )}}= \frac{(\nu +\varphi )(\nu _T+\theta )}{(\nu +\varphi )(\nu _T+\theta )-\varphi \theta } \end{aligned}$$

How much time does one spend in the untreated compartment? Well, we expect there to be $\frac{(\nu +\varphi )(\nu _T+\theta )}{(\nu +\varphi )(\nu _T+\theta )-\varphi \theta }$ visits, each lasting $\frac{1}{\nu +\varphi }$. Thus, if one starts in the untreated compartment, one expects to spend

52 $$\begin{aligned} \frac{(\nu +\varphi )(\nu _T+\theta )}{(\nu +\varphi )(\nu _T+\theta )-\varphi \theta } \cdot \frac{1}{\nu +\varphi }=\frac{ \nu _T+\theta }{(\nu +\varphi )( \nu _T+\theta )-\varphi \theta } \end{aligned}$$

much time there over the course of one’s lifetime. Looking back, we recognize this term as $V_{1,1}$. The other terms of *V* can be similarly understood.

There is a graph-theoretic way of approaching this interpretation as well (Brouwer et al. 2015). Let *A* be the matrix whose entries $a_{i,j}$ are the probabilities of moving from compartment *j* to compartment *i*; this is the adjacency matrix of the directed graph of the compartments, weighted by transition probability (or, depending on your definition of the adjacency matrix, its transpose). Then

53 $$\begin{aligned} A= \begin{bmatrix}0&{}\frac{\theta }{\nu _T+\theta } \\ \frac{\varphi }{\nu +\varphi }&{} 0\end{bmatrix}. \end{aligned}$$

Then,

54 $$\begin{aligned} I+A+A^2+A^3+\dots = (I-A)^{-1} \end{aligned}$$

is a matrix whose entries $m_{ij}$ give the expected number of visits to compartment *i* if one starts in compartment *j*. Thus, we can write $V^{-1}$ as the product of waiting times and expected number of visits.

55 $$\begin{aligned} \begin{aligned} V^{-1}&=\begin{bmatrix} \frac{1}{\nu +\varphi } &{}0 \\ 0 &{} \frac{1}{\nu _T+\theta }\end{bmatrix}(I-A)^{-1}\\&=\begin{bmatrix} \frac{1}{\nu +\varphi } &{}0 \\ 0 &{} \frac{1}{\nu _T+\theta }\end{bmatrix}\begin{bmatrix}\frac{ (\nu +\varphi )(\nu _T+\theta )}{(\nu +\varphi )( \nu _T+\theta )-\varphi \theta } &{} \frac{(\nu +\varphi ) \theta }{(\nu +\varphi )( \nu _T+\theta )-\varphi \theta }\\ \frac{ \varphi (\nu _T+\theta )}{(\nu +\varphi )( \nu _T+\theta )-\varphi \theta } &{}\frac{(\nu +\varphi )(\nu _T+\theta }{(\nu +\varphi )( \nu _T+\theta )-\varphi \theta } \end{bmatrix}\\&=\begin{bmatrix}\frac{ \nu _T+\theta }{(\nu +\varphi )( \nu _T+\theta )-\varphi \theta } &{} \frac{ \theta }{(\nu +\varphi )( \nu _T+\theta )-\varphi \theta } \\ \frac{ \varphi }{(\nu +\varphi )( \nu _T+\theta )-\varphi \theta } &{}\frac{\nu +\varphi }{(\nu +\varphi )( \nu _T+\theta )-\varphi \theta } \end{bmatrix}. \end{aligned} \end{aligned}$$

To complete the example, we calculate

56 $$\begin{aligned} \begin{aligned} K&=FV^{-1}= \begin{bmatrix} \beta &{} \beta _T \\ 0 &{} 0\end{bmatrix}\begin{bmatrix}\frac{ \nu _T+\theta }{(\nu +\varphi )( \nu _T+\theta )-\varphi \theta } &{} \frac{ \theta }{(\nu +\varphi )( \nu _T+\theta )-\varphi \theta } \\ \frac{ \varphi }{(\nu +\varphi )( \nu _T+\theta )-\varphi \theta } &{}\frac{\nu +\varphi }{(\nu +\varphi )( \nu _T+\theta )-\varphi \theta } \end{bmatrix}\\&= \begin{bmatrix}\frac{\beta (\nu _T+\theta )}{(\nu +\varphi )( \nu _T+\theta )-\varphi \theta } + \frac{\beta _T\varphi }{(\nu +\varphi )( \nu _T+\theta )-\varphi \theta } &{} \frac{\beta \theta }{(\nu +\varphi )( \nu _T+\theta )-\varphi \theta } + \frac{\beta _T(\nu +\varphi )}{(\nu +\varphi )( \nu _T+\theta )-\varphi \theta } \\ 0 &{}0 \end{bmatrix}, \end{aligned} \end{aligned}$$

so that

57 $$\begin{aligned} {\mathscr {R}}_0 = \frac{\beta (\nu _T+\theta )}{(\nu +\varphi )( \nu _T+\theta )-\varphi \theta } + \frac{\beta _T\varphi }{(\nu +\varphi )( \nu _T+\theta )-\varphi \theta }. \end{aligned}$$

Although we could simplify ${\mathscr {R}}_0$ to a single fraction, it aids interpretation to define the two terms as ${\mathscr {R}}_{0,I}$ and ${\mathscr {R}}_{0,T}$, respectively, so that ${\mathscr {R}}_0={\mathscr {R}}_{0,I}+{\mathscr {R}}_{0,T}$. Thus, ${\mathscr {R}}_0$ can be seen as the sum of terms, sometimes called submodel reproduction numbers, representing the contributions of untreated and treated individuals to the overall epidemic potential of the system. This type of additive structure is common in models with multiple infectious compartments. It can be interesting, for example, to understand how the relative contributions of ${\mathscr {R}}_{0,I}$ and ${\mathscr {R}}_{0,T}$ change as model parameters, e.g., the treatment $\varphi$ and relapse $\theta$ rate parameters, change.

## Conclusion

The basic reproduction number ${\mathscr {R}}_0$ is a fundamental and concept in mathematical epidemiology, and calculating ${\mathscr {R}}_0$ as a function of an infectious disease model’s parameters can improve one’s understanding of the disease system and the potential for disease control. In this paper, we developed intuition for the basic reproduction number of epidemic models both as an epidemiological construct and as a mathematical threshold. We used linear algebra and geometry to understand why the basic reproduction number is the spectral radius of the next generation matrix. We examined a series of simple models to see and interpret common patterns of parameter combinations. The intuition we developed here can be used when approaching more complex epidemic models.

## Data Availability

Data sharing is not applicable to this article as no datasets were generated or analyzed during the current study.
